# Comparison of GFAP and UCH-L1 Measurements from Two Prototype Assays: The Abbott i-STAT and ARCHITECT Assays

**DOI:** 10.1089/neur.2020.0037

**Published:** 2021-04-07

**Authors:** Frederick K. Korley, Saul A. Datwyler, Sonia Jain, Xiaoying Sun, Gangamani Beligere, Raj Chandran, Jaime A. Marino, Beth McQuiston, Hongwei Zhang, Krista L. Caudle, Kevin K.W. Wang, Ava M. Puccio, David O. Okonkwo, John K. Yue, Sabrina R. Taylor, Amy Markowitz, Geoffrey T. Manley, Ramon Diaz-Arrastia

**Affiliations:** ^1^ Department of Emergency Medicine, University of Michigan, Ann Arbor, Michigan, USA.; ^2^ Abbott Laboratories, Abbott Park, Illinois, USA.; ^3^ Herbert Wertheim School of Public Health, University of California, San Diego, La Jolla, California, USA.; ^4^ Abbott Point of Care, Ottawa, Ontario, Canada.; ^5^ U.S. Army Medical Materiel Development Activity (USAMMDA), Warfighter Brain Health Project Management Office (WBH PMO), Fort Detrick, Maryland, USA.; ^6^ Program for Neurotrauma, Neuroproteomics & Biomarker Research, Departments of Emergency Medicine, Psychiatry and Neuroscience, University of Florida, Gainesville, Florida, USA.; ^7^ Department of Neurological Surgery, University of Pittsburgh Medical Center, Pittsburgh, Pennsylvania, USA.; ^8^ Department of Neurological Surgery, University of California, San Francisco, San Francisco, California, USA.; ^9^ Brain and Spinal Injury Center, University of California, San Francisco, San Francisco, California, USA.; ^10^ Department of Neurology, University of Pennsylvania, Philadelphia, Pennsylvania, USA.; ^11^ Traumatic Brain Injury Clinical Research Center, Penn Presbyterian Medical Center, Philadelphia, Pennsylvania, USA.

**Keywords:** assay, biomarkers, glial fibrillary acidic protein, traumatic brain injury, ubiquitin carboxyl-terminal hydrolase L1

## Abstract

Glial fibrillary acidic protein (GFAP) and ubiquitin carboxyl-terminal hydrolase L1 (UCH-L1) may aid in the evaluation of traumatic brain injury (TBI). The objective of this analysis was to compare GFAP and UCH-L1 values measured using a handheld device compared with a core laboratory platform. We analyzed plasma samples from patients with TBI and healthy controls enrolled in the Transforming Research and Clinical Knowledge in TBI (TRACK-TBI) cohort study. GFAP and UCH-L1 were measured twice in each subject using prototype assays, first with the Abbott i-STAT™ handheld device, and second with the Abbott ARCHITECT^®^ platform. We then quantified the agreement in biomarker values obtained using these two methods. GFAP and UCH-L1 were measured twice in 570 and 572 samples, respectively. GFAP values measured by the ARCHITECT platform (median 143.3 [interquartile range (IQR): 19.8–925.8] pg/mL) were higher than values measured by the i-STAT (median 116.0 [IQR: 9.2–856.5] pg/mL). GFAP values from the two platforms were strongly correlated (*p* = 0.985). Similarly, UCH-L1 values measured by the ARCHITECT platform (median 163.9 [IQR: 82.5–412.4] pg/mL) were higher than values measured by the i-STAT (median 122.5 [IQR: 63.0–297.3] pg/mL). UCH-L1 values from the two platforms were strongly correlated (*p* = 0.933). Passing-Bablok regression equations were developed to estimate the relationship between the two platforms, specifically to predict i-STAT values from the ARCHITECT platform. GFAP and UCH-L1 values measured using the prototype assays on the Abbott i-STAT and ARCHITECT platforms are strongly correlated and values from either platform may be converted to the other.

## Introduction

Glial fibrillary acidic protein (GFAP) and ubiquitin carboxyl-terminal hydrolase L1 (UCH-L1) were recently cleared by the U.S. Food and Drug Administration (FDA) to aid in the evaluation of traumatic brain injury (TBI). A number of well-powered observational studies have demonstrated that these biomarkers have excellent diagnostic accuracy for identifying TBI with TBI who are likely to have traumatic intracranial abnormalities on head computed tomography (CT) scan.^1–4^ Abbott Laboratories has produced two prototype assays for measuring GFAP and UCH-L1; one a point-of-care i-STAT™ handheld device, and the other a core laboratory ARCHITECT^®^ platform. The prototype i-STAT handheld device can measure GFAP and UCH-L1 in whole blood, serum, and plasma with a turnaround time of 15 min.

The FDA recently provided 510(k) clearance for plasma GFAP and UCH-L1 measurement on the Abbott i-STAT. Similarly, the prototype ARCHITECT assay can measure GFAP and UCH-L1 in serum and plasma with a turnaround time of 18 min. However, it is not yet known whether GFAP and UCH-L1 values measured using these different platforms are commutable. This knowledge is important for informing the interpretation of literature reporting GFAP and UCH-L1 values using these platforms as well as for decisions regarding combining data from studies that used the two platforms. Accordingly, the objective of this study was to compare prototype GFAP and UCH-L1 values measured by the i-STAT and ARCHITECT platforms and to determine their degree of agreement.

## Methods

### Study population and data collection

We performed an analysis of plasma samples collected from patients enrolled in the Transforming Research and Clinical Knowledge in TBI (TRACK-TBI) cohort study. TRACK-TBI, the largest natural history of TBI in the United States, is a prospective observational study of persons evaluated for blunt TBI in emergency departments (EDs) of eight Level 1 trauma centers from February 26, 2014 to July 27, 2018.^5^ TRACK-TBI enrolled both patients with TBI and healthy control. TBI participants met the following criteria: 1) 0–100 years of age; 2) evaluated in the enrolling ED within 24 h of injury; 3) received a head CT scan as part of routine clinical care; 4) had adequate visual acuity/hearing pre-injury; 5) were fluent in English or Spanish. Healthy controls were non-injured persons who were either an existing family or friend of the TBI participant or were recruited through public advertisement within TRACK-TBI enrolling institutions. Healthy controls were excluded if they had a history of TBI, concussion, or any traumatic injury causing polytrauma in the 12 months before enrollment in the study.

Written informed consent was obtained from participants or a legally authorized representative. The study was approved by the institutional review boards (IRBs) of enrolling sites.

Blood samples were processed, aliquoted, and stored in a −80°C freezer within 2 h of draw until the time of biomarker measurement. Sample acquisition, processing, and storage were performed in accordance with the TBI-Common Data Elements (CDE) Biospecimens and Biomarkers Working Group Guidelines.^6^ Sample analysis occurred in a single laboratory (Abbott Laboratories, Abbott Park, IL, USA) and was conducted by personnel blinded to clinical information regarding participants.

To compare GFAP and UCH-L1 measurements across a wide range of values, we examined ethylenediamine tetraacetic acid (EDTA) plasma samples collected from both TBI and healthy control participants. A total of 375 samples from 344 TBI participants were selected from TRACK-TBI for inclusion in the study. We also studied a total of 197 samples from 181 healthy controls from TRACK-TBI.

### GFAP and UCH-L1 measurements

For each plasma sample, GFAP and UCH-L1 concentrations were measured twice, first on the i-STAT handheld device, and second on the ARCHITECT platform. Measurements on the i-STAT handheld device were performed between January 2017 and May 2018 and measurements on the ARCHITECT platform were performed between November 2019 and February 2020. Although GFAP and UCH-L1 may be measured in either serum or plasma, we selected plasma because processing plasma is faster than processing serum (no requirement to wait until samples clot). It is therefore the preferred medium for measuring biomarkers of acute injury. Assays utilized different aliquots of plasma obtained from the same subject. Samples were subjected to only one freeze-thaw cycle. Samples were centrifuged at 10,000 RCF for 10 min prior to testing.

The prototype i-STAT handheld device GFAP and UCH-L1 tests used the sandwich enzyme-linked immunosorbent assay (ELISA) method with electrochemical detection of the resulting enzyme signal. The test time for each assay was approximately 15 min. The prototype GFAP assay calibration range was 0–50,000 pg/mL. The limit of detection (LoD) and limit of quantitation (LoQ) were 15 pg/mL and 25 pg/mL, respectively, resulting in a reportable range of 15–50,000 pg/mL. Within-laboratory precision, measured by the co-efficient of variation (CV) was 2.8–14.2%. The prototype UCH-L1 assay calibration range was 0–20,000 pg/mL. The LoD and LoQ were 10 pg/mL and 20 pg/mL, respectively, resulting in a reportable range of 10–20,000 pg/mL. The assay had a CV of 5.0–10.0%. Samples were tested in duplicate in the i-STAT GFAP and UCH-L1 assays. Within-laboratory CV values are based on the results of 20-day precision studies following Clinical and Laboratory Standards Institute (CLSI) EP05-A3 guidance.^7^ i-STAT GFAP within-laboratory precision of 2.8–14.2% CV was demonstrated over a concentration range of 15,000–40 pg/mL. Higher CVs are observed as the concentration approaches the lower LoQ. The i-STAT UCH-L1 within-laboratory precision of 5.0–10.0% CV was demonstrated over a concentration range of 10,000–100 pg/mL.

The prototype ARCHITECT GFAP and UCH-L1 assays are two-step sandwich assays using chemiluminescent microparticle immunoassay (CMIA) technology. The prototype GFAP assay calibration range was 0–50,000 pg/mL. The LoD and LoQ were 2 pg/mL and 5 pg/mL, respectively, for a reportable range of 2–50,000 pg/mL. The within-laboratory CV was 2.0–5.6%. Samples with values >50,000 pg/mL were retested with a 10-fold automated dilution protocol. The prototype UCH-L1 assay calibration range was 0–25,000 pg/mL. The LoD and LoQ were 10 pg/mL and 20 pg/mL, respectively, for a reportable range of 10–25,000 pg/mL. The assay had a CV of 2.0–5.7%. Samples were tested in singlicate for the ARCHITECT GFAP and UCH-L1 assays.

For GFAP, a total of 187 samples (156 control and 31 TBI) had values below the LOD of the i-STAT and a total of 24 samples (17 control and 7 TBI) had values between the LOD and the LOQ of the i-STAT. No GFAP values were below the LOQ of the ARCHITECT. For UCH-L1, a total of 5 samples (3 control and 2 TBI) had values below the LOD of the i-STAT and a total of 6 samples (3 control, 3 TBI) had values between the LOD and the LOQ of the i-STAT. No UCH-L1 values were below the LOQ of the ARCHITECT. The raw values of these samples were utilized to minimize bias in our comparisons. Two observations in which GFAP >50,000 pg/mL were removed as they exceeded the upper limit of quantitation and were outside the reportable range.

### Statistical analysis

Medians and their corresponding interquartile ranges (IQRs) were used to summarize biomarker data, because they did not follow the normal distribution. We visualized the agreement between biomarker values obtained from the two platforms via scatter and Bland-Altman plots^8^ and quantified the correlation between the two assays using Spearman's correlation coefficient. Passing-Bablok regression^9^ was fit to determine the function to convert ARCHITECT to i-STAT handheld device values for GFAP and UCH-L1 separately. The 95% confidence intervals of the regression parameters were estimated using the bootstrap method. For comparison, we also modeled the conversion of ARCHITECT to i-STAT values using simple linear regression. Statistical analyses were conducted in R, version 3.6.1. We used the “mcr” package in R to conduct the Passing-Bablok regression.

## Results

The median age of the TBI subjects was 39 years (IQR: 26–57); the median age of healthy controls was 32 years (IQR: 25–50). Among TBI subjects, 76.7% (280) had a presenting Glasgow Coma Scale (GCS) score of 13–15 and 55.8% had a positive head CT scan. Additional details regarding the demographic and clinical characteristics of the study population are presented in Table 1.

**Table 1.** Characteristics of the Study Population

	TBI participants (*n* = 374)	Healthy controls (*n* = 197)
Median age in years (IQR)	39 (26–57)	32 (25–50)
Females (%)	94 (25.1)	95 (48.2)
Glasgow Coma Scale score		
3–8	59 (16.2)	
9–12	26 (7.1)	
13–15	280 (76.7)	
Positive head CT scan	206 (55.8)	
Disposition		
ED discharge	15 (4.0)	
Non-ICU admission	147 (39.3)	
ICU admission	212 (56.7)	

CT, computed tomography; ED, emergency department; ICU, intensive care unit; IQR, interquartile range; TBI, traumatic brain injury.

**FIG. 1.**
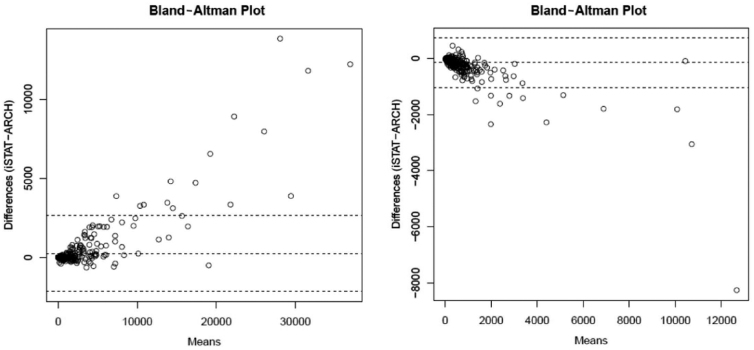
A Bland-Altman plot of GFAP and UCH-L1 levels. **(A)** Demonstrating that at higher GFAP values some outlier i-STAT™ handheld device values are much higher than ARCHITECT^®^ values. **(B)** Demonstrating that UCH-L1 values measured by the ARCHITECT^®^ are higher than values measured by the i-STAT™ handheld device throughout the range of quantifiable values. The dotted horizontal lines are the mean of the differences and corresponding 95% confidence intervals. GFAP, glial fibrillary acidic protein; UCH-L1, ubiquitin carboxyl-terminal hydrolase L1.

**FIG. 2.**
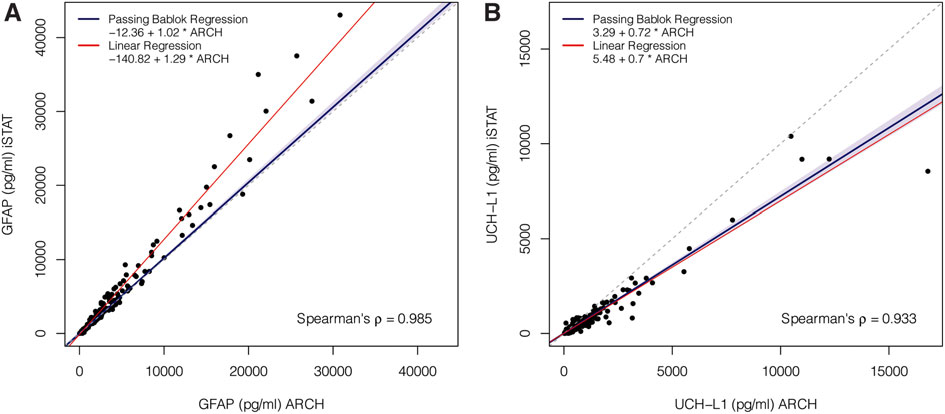
Scatter plot of GFAP and UCH-L1 values measured by the i-STAT™ handheld device and ARCHITECT^®^ assays. Shown are a scatter plot of GFAP **(A)** and UCH-L1 **(B)** values from the prototype Abbott i-STAT™ handheld device and prototype ARCHITECT^®^ assays. Shown are regression lines based on: 1) Passing-Bablok regression and 2) linear regression. GFAP, glial fibrillary acidic protein; UCH-L1, ubiquitin carboxyl-terminal hydrolase L1.

GFAP and UCH-L1 values were measured in 570 and 572 samples, respectively (Table 2). In the combined cohort of both TBI and healthy control participants, GFAP values measured by the ARCHITECT platform (median 143.3 [IQR: 19.8–925.8] pg/mL) were higher than values measured by the i-STAT handheld device (median 116.0 [IQR: 9.2–856.5] pg/mL); see Table 2 for median values for TBI participants only and healthy controls only. However, at higher GFAP values (e.g.: GFAP >5000 pg/mL) i-STAT handheld device values were higher than ARCHITECT values; see Figure 1A. GFAP values from the two platforms were strongly correlated (*p* = 0.985). Based on a Passing-Bablok regression model, i-STAT handheld device values may be estimated from ARCHITECT values using the equation i-STAT GFAP = −12.36 + (1.02 * ARCHITECT GFAP); see Figure 2A. The 95% confidence interval for the intercept was (−13.31 to −11.57) and (1.00 to 1.04) for the slope. The line fitted by the Passing-Bablok method was different from the line fitted by the simple linear regression because the non-parametric Passing-Bablok approach is more resistant to the effect of outliers.

**Table 2.** Summary of GFAP and UCH-L1 Values Measured by the Prototype ARCHITECT Platform and i-STAT Handheld Device

	Combined	Healthy controls	TBI participants
GFAP in pg/mL			
Median (IQR) ARCHITECT^®^	143.3 (19.8–925.8)	18.2 (14.0–25.5)	524.8 (153.5–2089.6)
Median (IQR) i-STAT™ handheld device	116.0 (9.2–856.5)	6.2 (2.1–13.4)	506.0 (128.5–2175.0)
Correlation co-efficient	0.985	0.854	0.993
UCH-L1 in pg/mL			
Median (IQR) ARCHITECT^®^	163.9 (82.5–412.4)	73.5 (56.2–104.8)	323.4 (146.7–751.7)
Median (IQR) i-STAT™ handheld device	122.5 (63.0–297.3)	62.0 (45.0–92.7)	206.0 (102.5–506.0)
Correlation co-efficient	0.933	0.795	0.947

GFAP, glial fibrillary acidic protein; IQR, interquartile range; UCH-L1, ubiquitin carboxyl-terminal hydrolase L1; TBI, traumatic brain injury.

Similarly, in the combined cohort of both TBI and healthy control participants, UCH-L1 values measured by the ARCHITECT platform (median 163.9 [IQR: 82.5–412.4] pg/mL) were higher than values measured by the i-STAT handheld device (median 122.5 [IQR: 63.0–297.3] pg/mL); see Table 2 for UCH-L1 values in TBI participants only and healthy controls only. This finding held true whether UCH-L1 was high or low (Fig. 1B). UCH-L1 values from the two platforms were strongly correlated (*p* = 0.933). Based on a Passing-Bablok regression model, i-STAT device values may be estimated from ARCHITECT values using the equation i-STAT UCH-L1 = 3.29 + (0.72 * ARCHITECT UCH-L1); see Figure 2B. The 95% confidence interval for the intercept was (−0.34 to 7.66) and (0.69 to 0.75) for the slope. The line fitted by the Passing-Bablok method was similar to the line fitted by simple linear regression because there were few outlier UCH-L1 values.

## Discussion

Our analyses demonstrate that both GFAP and UCH-L1 values measured using the prototype i-STAT handheld device assays are strongly correlated with GFAP and UCH-L1 values measured by the prototype ARCHITECT assays. The absolute differences in assay values between the two platforms become greater with increasing biomarker concentration. However, in general, values derived from the prototype ARCHITECT assay are higher than those derived from the prototype i-STAT handheld device assay. Nonetheless, given the strong correlation of the GFAP and UCH-L1 values measured by the two platforms, prototype ARCHITECT values may be converted to prototype i-STAT handheld device values using equations derived from Passing-Bablok regression.

Until recently, there were no blood-based biomarkers for diagnosing or monitoring acute brain injuries. In 2018, the FDA cleared the use of GFAP and UCH-L1 for aiding the evaluation of acute mild TBI. Their approval means that for the first time, clinicians may be able to use blood-based biomarkers to aid in diagnosing and risk-stratifying TBI. Given the time-sensitive nature of evaluating TBI and identifying patients with intracranial traumatic hemorrhage prior to clinical deterioration, point-of-care measurement of GFAP and UCH-L1 is attractive. An additional advantage of point-of-care assays is that they may be performed by trained clinical personnel and not necessarily by trained laboratory scientists. In addition, whole blood can be assayed directly, without centrifugation to obtain plasma or serum. However, core laboratory assays typically produce more analytically robust values, have a wider dynamic range, and have a higher overall throughput.^10^ As the literature on GFAP and UCH-L1 matures, it would be helpful to have a clear understanding on how values of these biomarkers differ when measured using different platforms. Conversion of ARCHITECT to i-STAT values will enable direct comparison of assay results derived from the two platforms. When converting ARCHITECT values to i-STAT values, derived i-STAT values that are below the LoD of the assay (15 pg/mL for GFAP and 10 pg/mL for UCH-L1) should be reported as undetectable.

Differences in values between the two assay platforms may be due to the different assay formats, calibration method, and technologies used by the i-STAT and ARCHITECT systems. For example, the i-STAT uses a one-step assay format where the sample is in contact with the detection and capture antibodies at the same time. The ARCHITECT uses a two-step format whereby the sample can bind to the capture antibody, and the mixture is then washed and followed by addition of the detection antibody. The differences in GFAP values for the control subjects (Table 2) may be due to differences in analytical sensitivity between the methods, where the ARCHITECT GFAP assay shows an improved LoD and LoQ compared with i-STAT. The i-STAT GFAP and UCH-L1 assays use approximately 20 μL of sample per test. The ARCHITECT GFAP assay uses 150 μL of sample per test and the ARCHITECT UCH-L1 assay uses 100 μL of sample per test.

Our findings have important implications with regards to interpreting the literature on these biomarkers. First, measured values of the same biomarker performed on the same blood sample using different platforms manufactured by the same company may not be numerically the same—although the assays in the present study demonstrated a strong correlation. Therefore, reference intervals and decision-making thresholds derived using different assays may be different, and as such, it would be helpful if articles reporting brain injury biomarker values also detail the analytic characteristics of assays. This will inform the interpretation of biomarker values across different studies.

The second is that there is sufficient scientific basis for combining GFAP and UCH-L1 values derived from the i-STAT handheld device and ARCHITECT platform. Individual biomarker studies with small sample sizes are often susceptible to the effects of chance, bias, and confounding factors. Meta-analyses of individual biomarker studies provide enhanced statistical power by combining all available results from similar studies that address a particular question, thereby reducing random error and increasing the precision in estimating the extent of the effect. They also enable the completion of subgroup analyses that may not be feasible in individual studies with small sample sizes. Findings from this study suggests that prototype ARCHITECT GFAP and UCH-L1 values may be estimated using prototype i-STAT handheld device values, enabling the combination of measurements derived from the two different platforms.

### Limitations

Our study has a number of limitations. First, the results of this analysis are applicable only to values obtained from the prototype i-STAT handheld device and ARCHITECT platform. Second, i-STAT values were generated using plasma samples. It is not known whether the correlation between i-STAT and ARCHITECT values will hold if whole blood is used for i-STAT measurements.

## Conclusion

GFAP and UCH-L1 values measured using the prototype Abbott i-STAT handheld device and prototype ARCHITECT assays are strongly correlated and values from either platform may be converted to the other.

## Notes

**Cite this article as:** Korley, FK, Datwyler, SA, Jain, S, Sun, X, Beligere, G, Chandran, R, Marino, JA, McQuiston, B, Zhang, H, Caudle, KL, Wang, KKW, Puccio, AM, Okonkwo, DO, Yue, JK, Taylor, SR, Markowitz, A, Manley, GT, Diaz-Arrastia, R, and the TRACK-TBI Study Investigators (2021) Comparison of GFAP and UCH-L1 measurements from two prototype assays: the Abbott i-STAT and ARCHITECT assays. *Neurotrauma Reports* 2:1, 193–199, DOI:10.1089/neur.2020.0037.

## Abbreviations Used

CDE: Common Data Elements
CLSI: Clinical and Laboratory Standards Institute
CMIA: chemiluminescent microparticle immunoassay
CT: computed tomography
CV: co-efficient of variation
ED: emergency department
EDTA: ethylenediamine tetraacetic acid
ELISA: enzyme-linked immunosorbent assay
FDA: U. S. Food and Drug Administration
GCS: Glasgow Coma Scale
GFAP: glial fibrillary acidic protein
ICU: intensive care unit
IQR: interquartile range
IRB: institutional review board
LoD: limit of detection
LoQ: limit of quantitation
TBI: traumatic brain injury
TRACK-TBI: Transforming Research and Clinical Knowledge in TBI
UCH-L1: ubiquitin carboxyl-terminal hydrolase L1
